# From standard to individualized diazoxide therapy in congenital hyperinsulinism: a narrative review

**DOI:** 10.3389/fphar.2026.1781424

**Published:** 2026-03-30

**Authors:** Teresa Wong, Daniel Chan, Cherie Chua, Wenqi Cher, Selina Lim, Suresh Chandran, Fabian Yap

**Affiliations:** 1 Department of Pharmacy, KK Women’s and Children’s Hospital, Singapore, Singapore; 2 Endocrinology Service, KK Women’s and Children’s Hospital, Singapore, Singapore; 3 Paediatrics Academic Clinical Program, DukeNUS Medical School, Singapore, Singapore; 4 Department of Paediatrics, KK Women’s and Children’s Hospital, Singapore, Singapore; 5 Department of Neonatology, KK Women’s and Children’s Hospital, Singapore, Singapore

**Keywords:** congenital hyperinsulinism, diazoxide, dosing, neonate, pharmacodynamic, pharmacokinetic

## Abstract

**Purpose:**

Congenital hyperinsulinism (CHI) is the commonest cause of persistent hypoglycaemia in neonates and infants (blood glucose <3.0 mmol/L in first 2–3 days of life; <3.5 mmol/L after 3 days of life). Diazoxide demonstrates variable efficacy depending on the underlying genetic variant and clinical phenotype. Diazoxide has been associated with side effects that are likely dose dependent. This narrative review synthesizes current evidence on diazoxide’s pharmacokinetics and side effect profile to support the development of individualised dosing strategies guided by genotype and patient-specific risk factors, with the aim of optimizing therapeutic outcomes and minimizing adverse effects.

**Methodology:**

A structured PubMed search was performed to review diazoxide use in hyperinsulinism (HI). Articles not involving neonatal HI, not specifying diazoxide dosing or focusing on alternative therapies were excluded. Addition articles were identified through reference screening or hand searched.

**Results:**

Traditional diazoxide dosing (5–15 mg/kg/day) is being challenged by emerging evidence supporting the benefits of lower starting doses (2–5 mg/kg/day), particularly in neonates with transient HI or specific genetic variants such as *HNF1A* and *HNF4A*. Lower starting doses in selected CHI patients have been shown to achieve adequate glycaemic control with fewer complications, including pulmonary hypertension (PH) and fluid retention. Diazoxide-responsiveness correlates strongly with the underlying molecular etiology. *ABCC8* and *KCNJ11* gene mutations often predict diazoxide-unresponsiveness, whereas *GLUD1*, *HADH*, and other variants typically predict responsiveness. Early predictors of unresponsiveness include large for gestational age (LGA) status (unrelated to maternal diabetes), early neonatal onset, and the need for high glucose infusion rates (GIR). Risk factors for adverse effects include renal impairment, prematurity, and low albumin levels, which may increase free diazoxide concentrations and drug toxicity.

**Conclusion:**

Individualized dosing based on clinical phenotype and genotype can improve safety and effectiveness in CHI management. Lower initial doses are recommended in patients with propensity for diazoxide sensitivity or risk factors for adverse events, while more rapid escalation may be warranted in suspected diazoxide-resistant cases.

## Introduction

Hyperinsulinism (HI) encompasses a heterogenous group of disorders characterized by inappropriate insulin secretion from pancreatic beta cells (β-cells) in presence of hypoglycaemia. Clinically, HI is broadly classified as transient or persistent forms. Transient neonatal HI can occur in infants of diabetic mothers (IDM), small for gestational age (SGA) infants with intrauterine growth restriction (IUGR), premature neonates or those with perinatal stress during delivery (e.g., perinatal asphyxia). Such transient forms are generally self-limiting, resolving within the first few weeks to months of life (Mohamed et al., 2012), but can also be severe in infants without risk factors (Yap et al., 2004). For consistency throughout this manuscript, the term HI will be used to encompass transient and persistent forms of hyperinsulinaemic hypoglycaemia (HH).

In contrast, persistent HI is frequently caused by an underlying genetic mutation, referred to as congenital hyperinsulinism (CHI) (Falzone et al., 2020; Shaikh et al., 2023; Banerjee et al., 2019), where the specific genetic variant involved is a key determinant of diazoxide-responsiveness. To date, mutations in at least 14 genes have been implicated in CHI (refer to Table 3), all of which plays a key role in regulating insulin secretion (Demirbilek et al., 2017). However, despite advances in genetic testing (Demirbilek et al., 2017), approximately half of CHI cases currently have no identifiable molecular cause, suggesting the presence of additional undiscovered genetic or epigenetic mechanisms. Identifying the underlying molecular etiology helps to prognosticate diazoxide-responsiveness and the natural evolution of disease.

In diazoxide-unresponsive cases or patients unable to tolerate diazoxide due to adverse effects, guidelines recommend 18F-fluoro-L-DOPA positron emission tomography (PET scan) in selected patients where genetic findings suggest potential focal disease (Shaikh et al., 2023; De Leon et al., 2024; Yorifuji et al., 2017). Focal lesions are most commonly associated with single paternally inherited variants in *ABCC8* or *KCNJ11*. Whereas mutations in genes such as *GCK* or *GLUD1* are typically associated with diffuse HI, in which PET scan is not indicated (States et al., 2021). Genetic testing may therefore prevent unnecessary scanning of infants with diffuse disease (refer to Table 3) (States et al., 2021). This distinction is clinically essential for guiding surgical management, as focal disease can be cured by a limited resection of the affected pancreatic region, whereas diffuse disease may require sub-total pancreatectomy when medical therapy fails (De Leon et al., 2024).

HI is a challenging condition to precisely diagnose and treat. Early recognition and prompt management of hypoglycaemia are crucial to prevent neuroglycopenic complications such as seizures, neurodevelopmental delay and irreversible brain damage. Diazoxide remains the first-line pharmacologic agent for CHI and the only FDA-approved pharmacologic treatment for CHI. However, its dosing requires careful consideration to balance efficacy with safety due to a spectrum of dose-dependent and idiosyncratic adverse effects. The most frequently reported side effects include hypertrichosis, fluid retention, oedema, gastrointestinal intolerance, and haematologic abnormalities such as neutropenia or thrombocytopenia (Herrera et al., 2018; Chen et al., 2021). More serious but less common complications include pulmonary hypertension (PH) and necrotizing enterocolitis (NEC), particularly among premature or low-birth-weight infants (Herrera et al., 2018; Prado et al., 2021; Pillai et al., 2025). Fluid retention and PH are of particular concern in neonates because of their potential to exacerbate respiratory distress or precipitate cardiac failure. Given these risks, diazoxide dosing must be individualized and initiated cautiously, with close clinical and hemodynamic monitoring to optimize glycaemic control while minimizing toxicity.

### Individualized dosing and evolving practices

Given the potential severity of diazoxide-related adverse effects, recent clinical practice has shifted toward a more cautious, individualized dosing approach. This evolution reflects a growing emphasis on individualized dosing–tailoring therapy to patient-specific genetic and clinical factors to maximize efficacy while minimizing toxicity.

**FIGURE 1:**
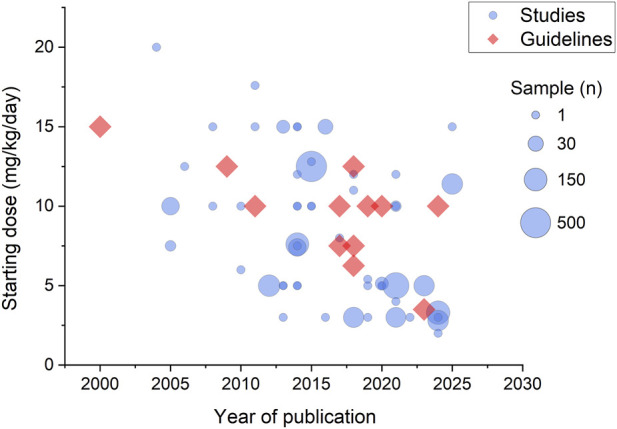
Trends in reported diazoxide dosing regimens over time for congenital hyperinsulinism.

Historically, diazoxide was dosed at 5–15 mg/kg/day (refer to Figure 1). However, recent studies suggest that lower starting doses (2–3 mg/kg/day) may be sufficient for patients with risk factors that predispose to diazoxide hypersensitivity such as *HNF1A* or *HNF4A* variants and SGA infants. The current evidence supporting this lower dose remains limited, consisting primarily of two single-patient case reports describing marked diazoxide sensitivity in individuals with *HNF4A* and *HNF1A* variants (Chandran et al., 2024; Arya et al., 2019), one small observational cohort study of SGA infants with HI (n = 56) demonstrating effective glycaemic control with mean effective doses below 5 mg/kg/day (Chandran et al., 2022), and a clinical correspondence reporting similar findings in routine practice (Ng et al., 2024). These early data suggests that low dose (≤5 mg/kg/day) diazoxide therapy may be as effective as traditional higher doses in managing hypoglycaemia in neonates with HI, while reducing hospitalization duration and incidence of adverse events, particularly in patients with genetic or clinical features associated with diazoxide hypersensitivity. However, larger prospective studies are required to validate these preliminary observations.

On the other hand, diazoxide-unresponsive patients often have malformed adenosine triphosphate (ATP)-sensitive potassium (K_ATP_) channels due to mutations in ATP Binding Cassette Subfamily C Member 8 (*ABCC8*) or Potassium Voltage-Gated Channel Subfamily J Member 11 (*KCNJ11*) genotypes (Banerjee et al., 2019). Earlier identification of diazoxide-resistance is essential to prevent risks of complications associated with higher diazoxide doses (Timlin et al., 2017).

Although expert consensus practice guidelines of diazoxide use in HI management have been published by the Pediatric Endocrine Society (PES) Drug and Therapeutics Committee work group (Brar et al., 2020), existing recommendations lack detailed guidance on individualized dosing strategies based on etiology or genotype. Our narrative review seeks to enhance current knowledge through further evaluation of individualized dosing (e.g., starting dose and titration of therapy) and examining how treatment can be tailored by genotype, clinical phenotype, and patient-specific risk factors. With growing insights from neonatal-onset HI and advances in molecular diagnostics, we aim to refine diazoxide dosing to optimize therapeutic outcomes while minimizing adverse effects.

## Methods

For this narrative review, a structured literature search was conducted on PubMed to identify articles evaluating diazoxide use in CHI (refer to Supplementary Figure S1). The search strategy combined Medical Subject Headings (MeSH) terms for diazoxide and CHI. The search was limited to English language publications from 1976 to 2025. Title and abstracts were screened for relevance. Full-text review was performed for eligible studies. Articles were excluded if diazoxide was not used in neonates or for HI management, no specific diazoxide data reported, diazoxide dose not specified, or if the articles focused on other management of HI. Additional articles were identified through manual screening of reference lists and hand search of relevant articles.

Given the heterogeneity of available data, findings were narratively synthesized with quantitative ranges reported where available.

## Diazoxide formulations and physicochemical properties

Diazoxide is available in multiple formulations, including tablets or capsules (25 mg, 50 mg, and 100 mg) and commercially manufactured oral suspension (50 mg/mL). The capsule was approved in the 1970s but due to its poor water solubility, precise dose adjustments with tablets or capsules can be challenging (Te va Pharmaceuticals USA and I., 2024). To improve dosing flexibility, especially in neonates and infants, an oral suspension was subsequently developed and approved in 1990. The suspension formulation is now preferred in paediatrics due to its ease of administration and better tolerability. In settings where the commercial 50 mg/mL oral suspension is unavailable, a 10 mg/mL extemporaneous suspension can be prepared by compounding the contents of diazoxide capsules into a liquid suspension. Stability studies have demonstrated that such preparations remain chemically stable for up to 90 days (Friciu et al., 2016). For infants requiring <10 mg per dose, the lower-concentration extemporaneous formulation may offer improved dosing accuracy compared to the higher-concentration suspension.

Although no direct comparative studies have evaluated the pharmacokinetics of diazoxide between the liquid and capsule formulations, the two formulations are considered bioequivalent (Te va Pharmaceuticals USA and I., 2024). The acid dissociation constant (pKa) of diazoxide in water is reported to be 8.5, and therefore at physiologic pH the drug remains largely unionized. This enhances its lipophilicity and membrane permeability, which is crucial for effective drug absorption (Pruitt et al., 1973).

## Pharmacokinetics

Little is known about diazoxide’s pharmacokinetic parameters in the neonatal population as pharmacokinetic studies in neonates are limited by ethical constraints, small blood volumes and clinical vulnerability. Consequently, pharmacokinetic data are generally extrapolated from adult or paediatric studies. Through established principles of neonatal developmental pharmacokinetics, we can infer likely trends in diazoxide pharmacokinetics for neonates.

The pharmacokinetics of diazoxide in paediatrics (median age 4.3 years, range 0.1–15.2 years) has been described by Kizu et al. using a one-compartment model, in which the volume of distribution (Vd) and clearance are proportional to body weight (n = 22) (Kizu et al., 2017). Kizu et al. demonstrated that similar steady-state concentrations were achieved in children receiving either twice-daily or thrice-daily dosing. The estimated time to reach steady-state concentration was 72–96 h for both dosing (Kizu et al., 2017). Thus, diazoxide was reported to be equally effective when administered twice or three times daily at an equivalent total daily dose. However, once-daily dosing resulted in significant fluctuations in drug levels and was deemed unsuitable (Brar et al., 2020; Kizu et al., 2017). Sex differences in diazoxide clearance have been observed, with females exhibiting a 39% greater clearance compared to males. However, this finding was derived from a small sample size and did not reach statistical significance, thus, no sex-specific dosing recommendations have been established (Brar et al., 2020). The maximal therapeutic response to diazoxide at the current dose should be assessed after at least 3 to 5 days of initiation or dose adjustments, after steady-state is achieved (Brar et al., 2020).

### Absorption

Diazoxide is a weak acid (pKa 8.74) and a lipophilic drug (log P 1.21) (PubChem Compound Summary for CID, 2026). Diazoxide is well absorbed orally, with a reported bioavailability of 85%–95% in adults (McInnes, 2007; Pearson, , 1977). Although time to maximum concentration (Tmax) have not been reported, onset of hyperglycaemic effect typically begins within 1 hour of oral administration according to drug monographs (Te va Pharmaceuticals USA and I., 2024). To our knowledge, diazoxide Tmax has not been systematically characterised in neonates or infants.

Diazoxide is primarily absorbed in the small intestine. While gastric pH may theoretically influence the ionisation of weakly acidic drugs, the clinical impact of transiently elevated gastric pH in neonates on diazoxide absorption is likely limited. Despite higher gastric pH in early life, diazoxide demonstrates good oral absorption in neonates, with bioavailability reported to be comparable to older children and adults (Ainsworth, 2020).

In contrast, developmental differences in gastric emptying are more likely to influence the rate rather than the extent of absorption. Neonates exhibit delayed gastric emptying during the first week of life, which may prolong Tmax and delay onset of action (Bansal et al., 2023). Gastric motility improves rapidly during the first week of life and is influenced by feeding practices, with human milk and lower-calorie feeds associated with faster gastric emptying compared to formula feeds (Bansal et al., 2023). Consequently, variability in onset of action may be observed in the early neonatal period, particularly during the first week of life.

### Distribution

The Vd of diazoxide following oral administration is 2 L (33% of body weight) in children with normal renal function (Pearson, 1977). At typical therapeutic concentrations, diazoxide is extensively and reversibly bound to about 90% of plasma proteins, mainly albumin (Pearson, 1977; Sellers and Koch-Weser, 1969; Pearson et al., 1976). Diazoxide exhibits a higher degree of plasma protein binding in adults compared to neonates due to the intrinsically lower albumin levels in neonates. This reduced protein binding in neonates results in a higher free drug concentration which may contribute to a shorter apparent half-life, although overall exposure remains strongly influenced by immature elimination pathways.

### Metabolism

Approximately 54%–60% of diazoxide undergoes metabolic transformation by the liver, while the remaining fraction is eliminated unchanged in the urine (Pearson, 1977; Pruitt et al., 1974). Diazoxide is primarily metabolized by CYP1A2, with a minor contribution from CYP3A4, and undergoes oxidation or sulfate conjugation at the methyl group to form inactive metabolites (Soleno Therapeutics, 2025; Newman-Lindsay et al., 2022). CYP1A2 activity is negligible at birth and undergoes post-natal induction, with progressive maturation over the first months of life and adult-like metabolic capacity reached by approximately 7–8 months of age (Bansal et al., 2023; O’Hara et al., 2015). Notably, CYP1A2 maturation correlates more closely with post-natal age than post-menstrual age (O’Hara et al., 2015). Therefore, hepatic metabolic clearance of diazoxide is expected to be limited in early life and rise gradually during infancy, potentially resulting in lower systemic exposure over time at a fixed weight-based dose.

### Excretion

Diazoxide is predominantly eliminated via the kidney, with 50%–80% excreted unchanged in the urine (Pruitt et al., 1973; Pearson, 1977; Pruitt et al., 1974; Newman-Lindsay et al., 2022; Lexicomp, 2025). In neonates, renal drug clearance is reduced due to immature glomerular filtration and tubular function, with more pronounced impairment in preterm and SGA infants (Bansal et al., 2023; O’Hara et al., 2015). Reduced glomerular filtration rate (GFR) at birth is therefore expected to further decrease diazoxide clearance, possibly resulting in higher systemic exposure early in life. GFR and tubular transporter activity increases rapidly in the first weeks of life (Bansal et al., 2023). In term neonates, adult levels of GFR are typically reached by approximately 2–3 months of age, whereas maturation may extend up to 1–2 years in preterm infants (Bansal et al., 2023). As renal function matures postnatally, diazoxide elimination is likely to rise accordingly with age.

### Integrated pharmacokinetic implications and clinical relevance

The half-life in neonates is not established in the literature. The pharmacokinetic study conducted by Kizu et al. obtained half-life of 15 ± 5.3 h (range 5.9–27.7 h) in children compared to 21–35 h in adults reported in other literature (Kizu et al., 2017; Sellers and Koch-Weser, 1969; Newman-Lindsay et al., 2022). According to drug monographs, the hyperglycaemic effects of diazoxide generally lasts no more than 8 hours in individuals with normal renal function (Te va Pharmaceuticals USA and I., 2024). This dissociation between the reported elimination half-life and the duration of action suggests that the pharmacodynamic effects of diazoxide are not solely dependent on circulating plasma drug concentrations.

Although reduced plasma protein binding in neonates may shorten the apparent half-life through increased unbound drug levels, overall diazoxide exposure in early life is predominantly governed by immature hepatic metabolism and renal excretion. The combination of immature hepatic metabolism and limited renal excretion at birth likely contributes to higher diazoxide exposure in the neonatal period, despite opposing effects of reduced protein binding on apparent half-life. These developmental changes may underlie the substantial inter- and intra-individual variability observed in diazoxide response during early infancy. Accordingly, ongoing dose optimisation based on weight gain may be required during early infancy, even in the absence of overt hypoglycaemia, particularly during the first months of life when renal and liver function matures rapidly. After approximately 8 months of age, once CYP1A2 activity approaches adult levels and renal function is well established, continued weight gain without dose escalation may result in a relative reduction in systemic exposure, and in some infants, this may allow for gradual reduction of weight-based dosing in the absence of recurrent hypoglycaemia.

A mechanistic understanding of age-dependent changes in diazoxide pharmacokinetics may assist clinicians in anticipating the need for dose adjustment over time and in interpreting variability in treatment response. However, given the multifactorial determinants of drug exposure in neonates, including maturation of hepatic and renal function, protein binding and feeding patterns, trends in drug levels are difficult to predict. Further prospective studies are required to establish pharmacokinetic–pharmacodynamic correlations that validate these developmental assumptions and inform evidence-based dosing strategies in the neonatal and early infant period.

### Effects of renal impairment on pharmacokinetics

Diazoxide use in patients with renal impairment warrants caution due to prolonged half-life that may require dose reduction (Brar et al., 2020). Studies have shown that the risk of hypotension correlates with increased severity of renal dysfunction (Pearson et al., 1976).

In patients with renal impairment, the proportion of unbound diazoxide is increased (Pearson et al., 1976). This is primarily due to reduced serum albumin levels in renal dysfunction, which lowers the extent of protein-bound diazoxide–reported at 83.9% in renally impaired patients compared to 94% in healthy adults (Pearson et al., 1976). Additionally, an inverse relationship between plasma urea levels and diazoxide binding has been observed. Higher plasma urea concentrations are associated with reduced protein binding of diazoxide, although this finding did not reach statistical significance (p = 0.08). Notably, elevated plasma urea levels were significantly correlated with a greater reduction in mean arterial blood pressure following diazoxide administration (p < 0.05) (Pearson et al., 1976).

Renal dose adjustment guidelines for diazoxide in children and neonates are lacking, and there are no published paediatric pharmacokinetic data or validated therapeutic drug monitoring strategies to guide dose titration in renal impairment. We suggest cautious use of diazoxide in renal impairment by initiating at lower doses, dose reduction or slower dose escalation because of reduced renal drug clearance. Closer monitoring of blood pressure to prevent hypotension, fluid balance and signs of oedema, full blood count and glucose levels is suggested to guide dose titration and avoid toxicity.

## Pharmacodynamics

Diazoxide’s therapeutic effect in HI is achieved by producing a dose-dependent increase in blood glucose, primarily through the inhibition of insulin release from the pancreatic β-cells, and to a lesser extent, via extrapancreatic mechanisms (Te va Pharmaceuticals USA and I., 2024). It acts by opening the K_ATP_ channels in pancreatic β-cell by binding to the sulfonylurea receptor 1 (SUR1) subunit. This increases potassium permeability across cell membrane leading to hyperpolarization of the β-cell membrane. Consequently, calcium influx is inhibited, resulting in reduced Ca^2+^-dependent insulin secretion (Chen et al., 2021; Xu et al., 2019; Coetzee, 2013; Hansen, 2006).

K_ATP_ channels are heterooctamers complexes composed of four pore-forming inwardly rectifying potassium channel subunits (Kir6.1 and/or Kir6.2), each coupled to a regulatory SUR subunit (Nichols, 2006). The SUR subunits are encoded by two genes: *ABCC8*, which encodes SUR1, and *ABCC9*, which encodes SUR2, with splice variants SUR2A and SUR2B (Coetzee, 2013).

The response of K_ATP_ channels to diazoxide depends on the specific subunit composition and intracellular adenosine diphosphate (ADP) levels (Matsuoka et al., 2000). Diazoxide strongly activates Kir6.2/SUR1 in the pancreatic β-cell channels and Kir6.2/SUR2B in smooth muscle channels under basal conditions with low ADP levels. In vascular smooth muscle, the activation of K_ATP_ channels induces vasodilation and contributes to its hypotensive effects (Coetzee, 2013). However, the hypotensive effect of diazoxide is more commonly observed with intravenous administration and is rare with oral doses used in HI. In contrast, activation of Kir6.2/SUR2A channels, typically found in cardiac and skeletal muscle, requires elevated cytosolic ADP concentrations (Coetzee, 2013). If activation occurs in pathologic conditions such as ischemia with raised ADP, it may lead to cardioprotective effects through action potential shortening and reduced myocardial excitability.

Diazoxide can cross the blood-brain barrier, allowing it to exert central effects (Kimonis et al., 2019; Miller et al., 2023). By activating K_ATP_ channels in neuropeptide Y (NPY)/agouti-related protein (AgRP) neurons within the hypothalamus, diazoxide reduces the secretion of potent appetite-stimulatory neuropeptides, and has been shown to attenuate hyperphagia (Miller et al., 2023; Cowen and Bhatnagar, 2020). Through these central mechanisms, diazoxide may yield improvements in hyperphagia, hepatic and circulating lipids and in insulin sensitivity (Kimonis et al., 2019). Additionally, diazoxide has been shown to enhance GABAergic activity, which may lead to reductions in aggressive and threatening behaviours in individuals with conditions such as Prader-Willi syndrome (Kimonis et al., 2019). However, this appetite-suppressing effect may affect euglycaemic control in neonates with HI, where diazoxide has often been associated with feeding difficulties and reduced appetite, due to its gastrointestinal and metabolic effects (Pillai et al., 2025; Pasquini et al., 2025). This highlights the need for close nutritional monitoring during diazoxide therapy, particularly in infants who are already at risk of poor feeding or growth failure.

Diazoxide exhibits several off-target pharmacological effects that could explain some of the associated adverse effects (refer to Table 1). One such effect is the activation of K_ATP_ channels in hair follicles, which prolongs the anagen phase of the hair cycle, resulting in hypertrichosis (Uno et al., 1990; Salido et al., 2013). Diazoxide also alters fluid and electrolyte homeostasis by reducing renal excretion of sodium, bicarbonate, and water, thereby expanding extracellular fluid volume (Herrera et al., 2018; Welters et al., 2015). This can result in hyponatremia, fluid retention, and peripheral oedema. Hyperuricemia may also occur due to impaired renal excretion of uric acid (Herrera et al., 2018).

**TABLE 1:** Adverse effects of diazoxide: frequency, dose, risk factors, and monitoring recommendations.

Adverse Effect	Frequency	Dose (mg/kg/day)	Onset	Risk factors	Reversibility	Monitoring (if any)	Ref
Hypertrichosis	45%–89%	Dose-independent Often >5	Unknown	No clear risk factors	Partially reversible upon dose reduction Potential treatment with topical sulfonylurea	Suggest for clinical monitoring at routine follow ups	Banerjee et al. (2019), Pasquini et al. (2025), Welters et al. (2015), Sharma et al. (2022), Newfield (2015), Pasquini et al. (2022)
Gastrointestinal symptoms (vomiting, nausea, poor appetite)	12%–58%	13.3 ± 8.2	Within 10 days	No known risk factors	Stopped 1–2 weeks after discontinuation of drug	Suggest for clinical monitoring at routine follow ups	Welters et al. (2015), Gong et al. (2016), Hu et al. (2012)
Hyponatremia and fluid retention/oedema	11%–30%	Dose dependent 12.3 ± 2.8, (range 8.5–17.5)	Within days	Lower gestational age; one or more PH risk factors	Reversible upon cessation	Combined treatment with a diuretic (hydrochlorothiazide) Monitor serum sodium level, fluid balance and weight	Herrera et al. (2018), Welters et al. (2015)
Hematological effects - Neutropenia - Thrombocytopenia	9%–15.6% 2%–4.7%	5–15	Within 28 days	No known risk factors	4–10 days	Suggest monitor full blood count at baseline, days 5–7 and then 3–6 monthly	Herrera et al. (2018)
Hyperuricemia	5%	5–15	Within 28 days	No known risk factors	510 days	Suggest monitor full blood count at baseline, days 5–7 and then 6 monthly	Herrera et al. (2018)
Pulmonary Hypertension (PH)	2.4%–6.2%	>10	Sudden onset after a high dosage or dose increase Mostly within 2 weeks (range: 6–50 days)	Prematurity, low BW, congenital heart disease	Reversible upon cessation	Echocardiography prior to initiation Fluid restriction to 130 mL/kg/day Immediate discontinuation if PH develops	Pasquini et al. (2025), Ohnishi et al. (2020), Thornton et al. (2019)
Necrotizing Enterocolitis (NEC)	0.9%–13% (case reports, small cohorts)	>10	5 days (range 1–12 days)	IUGR, SGA, Prematurity (33–36 weeks’ gestation) Diazoxide use within first week of life, Antenatal steroid use	Most resolved after discontinuation Cases of persistent NEC that led to death	Monitor for feeds intolerance, abdominal distension Consider restarting diazoxide only after NEC resolves, if hypoglycaemia persists	Prado et al. (2021), Gray et al. (2018)

Abbreviations: BW, birth weight; IUGR, intrauterine growth restriction; NEC, necrotizing enterocolitis; PH, pulmonary hypertension; SGA, small for gestational age.

In the gastrointestinal tract, diazoxide inhibits motility through smooth muscle hyperpolarization and inhibition of L-type calcium channels (Radulovic et al., 2015). Animal studies have found that diazoxide activates the KATP channels in the gut, which inhibits acetylcholine release, resulting in decreased smooth muscle contraction and reduced gastrointestinal motility (Zini et al., 1991). This can explain the gastrointestinal symptoms of poor appetite, nausea and vomiting reported in 12% of patients in a descriptive review (n = 644) (Welters et al., 2015) and the possible association between diazoxide and development of NEC in neonates suggested from a retrospective cohort study (n = 55) (Prado et al., 2021).

Additionally, diazoxide acts on mitochondrial K_ATP_ channels in pulmonary artery smooth muscle cells, leading to mitochondrial membrane depolarization, cytochrome C release, and hydrogen peroxide overproduction. This promotes cellular proliferation and inhibits apoptosis of the pulmonary artery smooth muscle cells, increasing pulmonary vascular resistance (Hu et al., 2010). Furthermore, diazoxide reduces inducible nitric oxide synthase activity, decreasing endogenous nitric oxide production which is crucial for vasodilation and maintaining pulmonary vascular tone by promoting smooth muscle cell relaxation (Yuan et al., 1998; Giaid and Saleh, 1995; Ohnishi et al., 2020). These combined effects results in increased pulmonary vascular resistance, which can contribute to the development of PH.

## Adverse events

Due to the above-mentioned off-target mechanisms, diazoxide is associated with a range of adverse effects, which vary in severity and clinical significance. The most frequently reported adverse effects include hypertrichosis (45%–89%), fluid retention (20%–30%), gastrointestinal disturbances (12%–58%), peripheral oedema (11%–25.7%), and neutropenia (9%–15.6%) (Chen et al., 2021; Pasquini et al., 2025). Less commonly, diazoxide has been linked to pulmonary hypertension (PH) (2.4%–6.2%) and thrombocytopenia (2%–4.7%) (De Leon et al., 2024; Herrera et al., 2018; Chen et al., 2021; Pasquini et al., 2025).

Among these, hypertrichosis, PH, and oedema warrant particular attention due to their prevalence and potential impact on patient outcomes. Hypertrichosis represents the most frequently observed side effect and appears to be largely independent of dosage (45%–89%). PH, though less common, is a recognized and serious adverse effect that has been highlighted by FDA, which may result in significant cardiovascular complications. Care must be taken in premature infants with chronic lung disease (CLD) as significant PH can lead to the reopening of the patent ductus arteriosus (PDA) (Kylat, 2019). Oedema and fluid retention can contribute to circulatory overload and exacerbate hemodynamic instability. For neonates on high glucose infusion rate (GIR) with cardiac risk factors, it is advisable to start diazoxide at a low dose and gradually increase the dose over the first few days with close attention to the fluid balance. A diuretic such as hydrochlorothiazide should be added to all patients starting on diazoxide to reduce risk of fluid retention as recommended by most guidelines (Shaikh et al., 2023; De Leon et al., 2024; Yorifuji et al., 2017; Brar et al., 2020; Welters et al., 2015).

Hematological effects, such as thombocytopenia and neutropenia, are typically mild and transient, without clinically significant risk of infection or bleeding (Herrera et al., 2018). Rarely, case reports have reported more severe thrombocytopenia with significant bleeding symptoms such as in a case of a 2.5 year old boy with transient CHI after dose increment of diazoxide to 10 mg/kg/day (Thus et al., 2019). There was also a reported case of HI/HA syndrome due to a heterozygous mutation of *GLUD1* who developed diazoxide associated neutropenia after 8 years of diazoxide use after dose was increased from 8 mg/kg/day to 12 mg/kg/day. Subsequently, a lower dose of diazoxide (6 mg/kg/day) successfully controlled hypoglycemia and cell counts increased even though they were not normalized. Neutropenia in the current case again improved in the period when the patient discontinued the drug use at her own will, proving a dose-dependent relationship (Vuralli et al., 2021).

Gastrointestinal disturbances, such as nausea vomiting and poor appetite, are more common at higher doses. Rarely, case reports of NEC have been reported, often associated with doses >10 mg/kg/day (Değirmencioğlu et al., 2013; Doheny et al., 2014; Keyes et al., 2021; Duggal et al., 2024).

### Suggested baseline investigations and monitoring of side effects

Baseline investigations should include a complete blood count, renal panel including serum electrolytes, serum albumin (as diazoxide is highly-albumin bound) and an echocardiogram to rule out structural heart defects and PH, as well as a baseline weight before diazoxide initiation. A re-check of these blood investigations and clinical monitoring of fluid status/oedema should be performed when increasing doses of diazoxide or based on clinical findings. At Children’s Hospital of Philadelphia Congenital Hyperinsulinism Center, they suggest for routine complete blood count and uric acid levels approximately 2–3 months after initiation of diazoxide and every 6 months thereafter (Herrera et al., 2018). We suggest for clinical monitoring, including checking in with patients/caregivers on these potential side effects of hypertrichosis and gastrointestinal symptoms at routine follow-ups.

### Pulmonary hypertension (PH)

With increasing reports of PH developing in infants, the FDA issued a drug safety communication warning of this association (Timlin et al., 2017; FDA, 2015). The frequency of diazoxide-related PH has been reported to be 2.4%–6.2% across retrospective cohorts (De Leon et al., 2024; Herrera et al., 2018; Pasquini et al., 2025; Ohnishi et al., 2020; Thornton et al., 2019).

There is little consistency in the time to onset of diazoxide-related PH and remains unclear whether these symptoms develop slowly over time (Timlin et al., 2017). However, PH generally exhibits a sudden onset, especially following high dosages or after a dose increment (Ohnishi et al., 2020). According to a retrospective multicentre study by Chen et al., 61.5% (8 out of 13) of patients who developed PH did so within 2 weeks of diazoxide initiation (Chen et al., 2019). The onset of PH may vary from 6 to 50 days after initiating treatment (Pillai et al., 2025).

PH most commonly occurred in patients treated with diazoxide doses greater than 10.4–11.5 mg/kg/day and was less likely when doses were below 10 mg/kg/day (Timlin et al., 2017; Ohnishi et al., 2020). The dose of diazoxide was found to be correlated with the severity of PH (Ohnishi et al., 2020).

The manufacturer recommends discontinuing diazoxide if PH is suspected (Te va Pharmaceuticals USA and I., 2024). Complete diazoxide withdrawal led to PH resolution at 41 days (Chen et al., 2019). It was found that dose reduction did not lead to resolution of PH when decreased from 12 mg/kg/day to 8 mg/kg/day. PH persisted and only resolved after complete discontinuation of diazoxide. It remains unclear whether PH would have fully resolved with more time at the lower dose (Timlin et al., 2017).

Risk factors for PH include prematurity, low BW or SGA, respiratory distress or CLD (e.g., pulmonary hypoplasia, bronchopulmonary dysplasia), and in infants with perinatal stress-induced hyperinsulinism (PSHI) which is a subtype of transient HI (Herrera et al., 2018; Pillai et al., 2025). Perinatal risk factors include maternal hypertension or preeclampsia and IUGR. Cardiac risk factors include congenital heart disease, cardiomyopathy and structural heart anomalies, such as PDA, cyanotic congenital heart disease, left heart obstruction, intracardiac shunt lesions, and pulmonary vein stenosis (Herrera et al., 2018; Pillai et al., 2025). Additionally, a total fluid volume exceeding 130 mL/kg/day in the 24 h prior to diazoxide initiation increases the risk of PH (Chen et al., 2019). Therefore, some authors and guidelines recommend to restrict fluid intake in the neonatal period to 130–150 mL/kg/day prior to and while ongoing diazoxide treatment to reduce the risk of pulmonary overload (Shaikh et al., 2023; Banerjee et al., 2019; Brar et al., 2020).

## Dosing considerations for diazoxide in congenital hyperinsulinism (CHI)

The therapeutic aim in CHI is to maintain euglycaemia and prevent undesirable consequences of recurrent hypoglycaemia, especially on neurodevelopment. First-line management prioritizes nutritional support through continuous or frequent feeding, with intravenous glucose as needed. In infants with risk factors for transient HI—such as those born SGA, IDM, or with perinatal asphyxia—pharmacologic treatment may not be necessary if euglycaemia can be achieved with watchful waiting while providing nutritional support and glucose infusion (Chandran et al., 2023). Transient HI tends to resolve spontaneously within the 6 months (Brar et al., 2020).

Diazoxide should be initiated in infants who require a high GIR to maintain euglycaemia or are unable to transition to 3–4 hourly feeds due to recurrent hypoglycaemia. In such cases, guidelines from the United Kingdom National Collaborative Consensus (Shaikh et al., 2023), the International Consortium of Paediatric Endocrinology (ICPE) (De Leon et al., 2024) and the Japanese Societies of Paediatric Endocrinology and Paediatric Surgeons (Yorifuji et al., 2017), recommend starting diazoxide at a therapeutic range of 5–15 mg/kg/day given in two or three divided doses.

**FIGURE 2:**
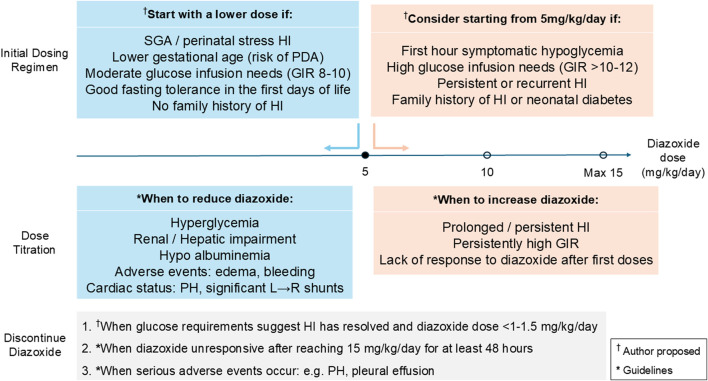
Pragmatic framework for diazoxide initiation, titration, and discontinuation in neonatal hyperinsulinaemic hypoglycaemia. Abbreviations: GIR, Glucose infusion rate; HI, Hyperinsulinism; PDA, Patent ductus arteriosus; PH, Pulmonary hypertension; SGA, Small for gestational age.

The glycaemic effect of diazoxide typically begins within an hour of administration. In practice, dose titration can be performed cautiously after first doses to achieve euglycemia and prevent prolonged hypoglycaemia (refer to Figure 2). However, definitive assessment of maximal efficacy at current dose should be made only after steady-state levels are achieved, typically 3 to 5 days after its initiation. Failure to reduce glucose infusion requirements by this time, despite receiving 15 mg/kg/day indicates diazoxide-unresponsiveness (Yorifuji et al., 2017; Kizu et al., 2017). In such cases, drug discontinuation should be considered as there is no evidence that exceeding this dose improves clinical outcomes, and doing so may expose patients to a greater risk of side effects.

The therapeutic dosing of diazoxide in HI requires individualized precision, as adverse effects such as fluid retention have been reported even in patients who are given lower doses (Herrera et al., 2018; Welters et al., 2015). The efficacy and adverse effects of diazoxide vary significantly between individuals, depending on severity and duration of HI, underlying etiology, gestational age and BW. Given this variability, treatment should begin with the lowest possible dose, balancing efficacy and safety (refer to Figure 2). While some protocols recommend starting therapy at 5 mg/kg/day and adjusting upwards, others may begin at 10–15 mg/kg/day (Brar et al., 2020) and titrate down if needed. For certain groups such as SGA or those with milder forms of the disease, emerging evidence suggests that starting with doses lower than the conventional 5–15 mg/kg/day may still be effective while reducing the risk of adverse effects. Figure 2 combines guideline- and consensus-derived elements (including dose ranges, maximum dose of 15 mg/kg/ day, and indications for discontinuation) with author-proposed clinical decision guidance on early risk stratification, and dose escalation, informed by observational data and tertiary- center practice.

Response to diazoxide is defined as sustained reduction in GIR, avoidance of hypoglycaemia, and/or improved age-appropriate fasting tolerance, assessed over 24-48 h following initiation or dose adjustment. The framework is intended to support clinical decision-making rather than to function as a prescriptive algorithm.

### Evidence of low dose diazoxide use

Several small retrospective studies and case series have evaluated the efficacy of lower starting doses of diazoxide (≤5 mg/kg/day), particularly in neonates with transient HI or PSHI. For example, Chandran et al. showed that in a cohort of 27 SGA infants with HI who required treatment with diazoxide, the mean and median effective dose of diazoxide was 4.6 and 4 mg/kg/day respectively; the authors recommended a starting dose of 3 mg/kg/day and titrating upwards as required to this particularly sensitive cohort of patients (Chandran et al., 2022). In a subsequent study of 73 infants, Malhotra et al. initiated diazoxide at 2, 3 and 5 mg/kg/day for infants according to BW bands of 1000g–2000g, 2000g–3500g and >3500g respectively, and found that 65% of the infants did not require dose escalation and the median effective dose was 3 mg/kg/day (Malhotra et al., 2024). While their classification of HI was made in hindsight, the findings suggest that initiating treatment with lower diazoxide doses at 2–3 mg/kg/day may be sufficient in a substantial proportion of cases. A third study by Ng et al. supports the use of low dose diazoxide in HI among SGA and non-SGA infants (n = 34), showing that discharge doses remained at or below 5 mg/kg/day in all infants, with only 17.6% requiring minimal up-titration during follow up–still within the low dose range–highlighting its effectiveness in routine practice regardless of gestation or BW (Ng et al., 2024).

There are also specific instances where even lower, or ultra-low, doses of diazoxide are necessary–particularly in certain forms of CHI. For example, case reports of a patient with *HNF4A* variants, as described by Arya et al. (2019), and another with *HNF1A* variant, as reported by Chandran et al. (2022), exhibited marked hyperglycaemia when treated with conventional doses such as 5 mg/kg/day. In these cases, effective glycaemic control was achieved with much lower doses of between 0.7 and 1.5 mg/kg/day, underscoring the importance of genotype guided dosing. A summary of outcomes on the use of lower diazoxide doses in selected CHI patients can be found in Table 2.

**TABLE 2:** Low dose diazoxide use in selected patients with hyperinsulinemia.

Study author and year	Patient population	Diazoxide initiation dose	Diazoxide effective dose	Adverse events reported
Chandran et al. (2022)	56 preterm and term SGA infants with HH	3 mg/kg/day in two divided doses, median age of initiation at day 10 of life	Mean 4.6 ± 2.2 mg/kg/day	Generalised hypertrichosis occurred in 2 patients (7.4%) that were on 10 mg/kg/day; fluid retention in 1 infant (3.7%)
Malhotra et al. (2024)	73 infants with transient HI (<6 months duration)	Depending on birth weight 1000g–2000 g: 2 mg/kg/day 2000g–3500 g: 3 mg/kg/day >3500 g: 5 mg/kg/day	Mean 3 mg/kg/day (range 1.5–10) 35% (26/73) required an increase from the starting dose	All adverse events (18%); oedema (12%) and hyponatraemia (5%) were the most common 2 infants developed suspected NEC; none had pulmonary hypertension
Ng et al. (2024)	34 infants with biochemically confirmed HH; 15 SGA, 19 non-SGA, 2 with genetically proven *HNF4A* mutation	<5 mg/kg/day for all cases	SGA preterm: 3 mg/kg/day SGA term: 2.74 mg/kg/day Non-SGA preterm: 2.48 mg/kg/day Non-SGA term: 3 mg/kg/day	5 infants (14.7%) were identified to have minimal fluid retention during follow‐up which resolved on increasing the dose of diuretics
Arya et al. (2019)	Term female infant, birth weight appropriate for gestational age (AGA), with maternally inherited *HNF4A* heterozygous nonsense mutation (p. Ser419Ter; c.1256C>G)	5 mg/kg/day	1.5 mg/kg/day	Marked hyperglycaemia (blood glucose 21.6 mmol/L) with initial dose of diazoxide, necessitating temporary cessation of diazoxide
Chandran et al. (2024)	Term male infant, birth weight large for gestational age (LGA), with maternally inherited *HNF1A* heterozygous mutation (p.Gly355Val; c.1064G>T)	5 mg/kg/day	0.7 mg/kg/day	Hyperglycaemia (blood glucose 16.6 mmol/L) within 48 h of diazoxide initiation, necessitating temporary cessation of diazoxide

Abbreviations: AGA, appropriate for gestational age; HH, hyperinsulinemia hypoglycaemia; LGA, large for gestational age; NEC, necrotising enterocolitis; SGA, small for gestational age.

However, these studies are limited by small sample sizes, single-center design and heterogeneity in definitions of responsiveness. Importantly, no randomized controlled trials have directly compared low-dose versus standard-dose initiation strategies, and current evidence is derived from observational studies.

When initiating diazoxide, based on institutional clinical expertise and experience, we recommend an individualized approach when deciding on a starting dose that aims to optimize the balance between therapeutic benefit and risk, based on unique patient-specific factors. It should consider patient’s risk factors for HI, clinical comorbidities and genetic results. Based on these observational studies, a lower starting dose of 3–5 mg/kg/day may be considered for infants with classic risk factors for transient HI (e.g., IUGR, SGA, perinatal asphyxia or IDM), as well as those with comorbidities that increase the risk of diazoxide-related adverse effects, including PH, cardiac failure, oedema, hyponatremia, or NEC. Dose escalation should include careful consideration of patient factors, including those with co-existing renal or liver impairment, cytopenia, sepsis or cardiovascular disease. This approach is supported by limited retrospective observational studies and clinical practice experience rather than high-quality comparative trials.

When genetic results are available–or strongly suspected based on family history–diazoxide dosing should be guided by the expected responsiveness associated with the identified variant (refer to Table 3). Lower doses of diazoxide may be used in those with *HNF4A* variants, while dose escalation may be more rapid for those with autosomal recessive *ABCC8* mutations.

**TABLE 3:** Genetic variants in congenital hyperinsulinism and their mechanisms of insulin dysregulation (Shaikh et al., 2023; Banerjee et al., 2019; Demirbilek and Hussain, 2017; Wabitsch et al., 2007).

Gene involved	Protein encoded	Mechanism	Inheritance	Clinical Characteristics	Diazoxide-Responsiveness
*ABCC8*	SUR1 subunit of K_ATP_ channel	Loss of function mutations may inhibit trafficking of SUR1 to the plasma membrane, or inhibit channel activity	AR, AD	Focal pancreatic lesions in cases with single paternally inherited variants Diffuse disease in biallelic recessive or monoallelic dominant (States et al., 2021)	Unresponsive in homozygous recessive or compound heterozygous cases; Responsive in dominant cases
*KCNJ11*	Kir6.2 subunit of K_ATP_ channel	Loss of function mutations may inhibit trafficking of Kir6.2 to the plasma membrane, or inhibit channel activity	AR, AD		
*GLUD1*	Glutamate dehydrogenase (GDH), a mitochondrial matrix enzyme	GDH catalyzes oxidative deamination of glutamate to alpha-ketoglutarate and ammonia, and is activated by leucine and inhibited by guanosine triphosphate (GTP) GLUD1 mutations decrease sensitivity to GTP inhibition, hence gain of function of GDH, resulting in leucine induced post prandial HI and high ammonia levels	AD	Often diffuse disease (States et al., 2021) Both fasting and leucine (protein) induced post-prandial HI occur, alongside raised plasma ammonia and urinary alpha-ketoglutarate Seizures may occur regardless of severity of hypoglycemia	Responsive
*HADH*	L-3-Hydroxyacyl-CoA Dehydrogenase, a mitochondrial enzyme	Loss of function mutations impair the enzymatic inhibitory effect of HADH on GDH, resulting in inappropriate leucine-sensitive HI and high ammonia levels	AR	Variable presentation ranging from severe neonatal HI to mild, late (adult) onset, leucine (protein)- induced HI. Normal plasma ammonia levels. May have elevated urinary 3-hydroxy-butyryl-carnitine and urinary 3-hydroxy-glutaric acid	Responsive
*SLC16A1*	Monocarboxylate transport (MCT1)	Gain of function mutations leads to increased expression of MCT1, which promotes transport of pyruvate and lactate across β-cell membrane, which enters Kreb’s cycle and stimulate insulin secretion in states of low plasma glucose during anaerobic exercise	AD	Hypoglycemia particularly triggered by strenuous exercise	Usually responsive; Some may demonstrate partial response (Galcheva et al., 2019; Tosur and Jeha, 2017; Otonkoski et al., 2003)
*HNF-1A*	HNF-1A transcription factor	Loss of function mutations result in dysfunctional nuclear hormone receptors and in turn impair the regulation of glucose-dependent insulin secretion from the β-cells	AD	HI is often transient. Patient can develop MODY later in life and this variant should be considered if there is family history of early diabetes	Responsive
*HNF-4A*	HNF-4A transcription factor		AD	Macrosomia at birth HI is often transient. Patient can develop MODY later in life and this variant should be considered if there is family history of early diabetes	
*GCK*	Glucokinase	Gain of function mutations result in increased affinity for glucose and reduced threshold for glucose-stimulated insulin release	AD	Often diffuse disease (States et al., 2021) Variable expression and phenotype even within the same family	May be diazoxide-responsive or unresponsive
*UCP2*	Mitochondrial Uncoupling Protein 2	Loss of function heterozygous mutations result in increase in intracellular ATP synthesis and enhanced glucose oxidation, with resultant glucose-mediated insulin secretion	AD	Not specifically described	Usually responsive (Ferrara et al., 2017; Gϋemes et al., 2020)
*HK1*	Hexokinase 1	Gain of function mutation results in increased phosphorylation of glucose to glucose-6-phosphate and inappropriate insulin secretion at low plasma glucose levels	AD	Not specifically described	Uncertain
*PMM2*	Phosphomanno-mutase 2	PMM2 is an enzyme involved in glycosylation and insulin secretion from β-cells	AR	Large for gestational age at birth, Polycystic kidneys, Polycystic liver	Responsive (Chen and Sang, 2022)
*PGM1*	Phosphogluco-mutase 1	PGM1 is an enzyme that catalyzes reversible conversion of glucose-6-phosphate to glucose-1-phosphate involved in glycogen metabolism. A loss of function mutation results in exaggerated insulin secretion	AR	Fasting and post-prandial HI. May be associated with short stature, cleft palate, myopathy (Galcheva et al., 2019; Stanley, 2016)	Uncertain
*FOXA2*	Forkhead Box Protein 2	Transcription factor implicated in the pancreas development and required for notochord formation during embryonic development involved in endoderm-derived organ system. Mechanism not fully understood	Sporadic	Pituitary gland defects, pituitary hormone deficiency (Vajravelu et al., 2018)	Uncertain
*CACNA1D*	L-type voltage-gated calcium channel	Activating mutation results in voltage-gated calcium channels remaining open at lower membrane potential and dysregulation of insulin secretion. Mechanism not fully understood	AD	Cardiac defects, hypotonia (Flanagan et al., 2017)	Uncertain

Abbreviations: AD, autosomal dominant; AR, autosomal recessive; ATP, adenosine triphosphate; GDH, glutamate dehydrogenase; GTP, guanosine triphosphate; HI, hyperinsulinism; K_ATP_, ATP-sensitive potassium; MCT1, monocarboxylate transport; MODY, maturity onset diabetes of the young; SUR, sulfonylurea receptor.

Discontinuation of diazoxide may be considered under several clinical scenarios (refer to Figure 2). First, in cases where HI has resolved–indicated by sustained euglycemia while on a diazoxide dose of <1 mg/kg/day (Yorifuji et al., 2017). Second, if the patient is deemed diazoxide-unresponsive, evidenced by continued dependence on intravenous glucose despite receiving 15 mg/kg/day of diazoxide after steady-state is achieved (De Leon et al., 2024; Yorifuji et al., 2017). Lastly, treatment should be discontinued in the event of serious adverse effects, such as the development of PH (refer to Table 1) (Te va Pharmaceuticals USA and I., 2024; Chen et al., 2019).

## Factors predicting response to diazoxide

Diazoxide-responsiveness in children with HI varies significantly and is largely influenced by the underlying etiology and pathophysiology. Multiple factors have been identified as determinants of diazoxide response, including the type of HI (transient vs. persistent), specific genetic variants involved, associated syndromic conditions and patient characteristics such as the age of symptom onset and BW.

### Diazoxide-responsiveness in transient hyperinsulinism (HI)

Some newborns develop transient HI due to presence of perinatal stress, such as being SGA, IUGR, perinatal asphyxia or IDM. In the absence of other genetic abnormalities, this group of patients are more likely to be diazoxide-responsive (Shaikh et al., 2023; Brar et al., 2020). Infants who are born SGA with or without IUGR and those who experience birth asphyxia have inappropriately high levels of insulin as a result of disruption in the regulation of insulin synthesis, which is often temporary and resolves on subsequent clinical follow-up (Falzone et al., 2020; Shaikh et al., 2023). IDM have higher levels of insulin due to intrauterine exposure to high blood glucose levels, and their insulin levels gradually normalize over the postnatal period. Hence, these patients often have transient HI and some may not require diazoxide therapy if euglycaemia can be maintained without intravenous glucose support after 7–10 days of life (Brar et al., 2020). In a retrospective study of 141 neonates with transient HI, only 24% required diazoxide treatment (Davidov et al., 2020). Similarly, a large retrospective cohort study analyzing data from 392 neonatal intensive care units between 1997 and 2016, reported that among 185,832 infants with hypoglycaemia, only 1,066 (0.57%) required initiation of diazoxide treatment (Gray et al., 2018).

Others who take a longer duration to recover are likely to benefit from diazoxide (Newman-Lindsay et al., 2022; Yoshida et al., 2014). Davidov et al. reported that those who eventually require treatment with diazoxide (n = 34) tend to have higher mean C-peptide levels detected during hypoglycaemia (1.4 ± 0.9 vs. 0.8 ± 0.5 ng/mL) and require higher GIR (16.6 ± 3.4 vs. 10.4 ± 4.0 mg/kg/min) to maintain normoglycaemia (Davidov et al., 2020).

### Diazoxide-responsiveness in relation to underlying genetic variants

Patients with persistent HI commonly have an underlying genetic mutation (Falzone et al., 2020; Shaikh et al., 2023; Banerjee et al., 2019) and affects one in 13,500 to 45,000 newborns in non-consanguineous populations (Hewat et al., 2022). Genetic studies have identified congenital mutations in 14 genes (refer to Table 3) that play a key role in regulating insulin secretion resulting in HI and the specific genetic variant involved is a key determinant of diazoxide-responsiveness (Demirbilek et al., 2017). Of these, the most common genetic mutation is due to defects in genes encoding the K_ATP_ channel and they are both located on chromosome 11p15.1. The K_ATP_ channel consists of two protein subunits; the SUR1 and Kir6.2, which are encoded by the *ABCC8* and *KCNJ11* gene respectively.

The identification of the genetic variant and the underlying molecular mechanism of CHI helps to prognosticate diazoxide-responsiveness and the natural evolution of disease. A retrospective cohort study (n = 300) by Kapoor et al. identified a genetic mutation in 87.6% of those with diazoxide-unresponsive CHI, in contrast to only 22.4% of patients with diazoxide-responsive CHI (Newman-Lindsay et al., 2022; Kapoor et al., 2013).

Table 3 shows the spectrum of genes that have been reported to cause CHI thus far, and the mechanisms by which they result in insulin dysregulation. Diazoxide is generally effective when K_ATP_ channel function is preserved. Patients with mutations in *GLUD1*, *HADH*, *SLC16A1*, *HNF1A* and *HNF4A* genes are more likely to be diazoxide-responsive as compared to the other genetic variants (Shaikh et al., 2023; Kapoor et al., 2013; Demirbilek and Hussain, 2017; Flanagan et al., 2010). *HNF1A* and *HNF4A* variants can present with transient or prolonged HI in infancy and diabetes in adolescence and should be suspected if there is a family history of maturity-onset diabetes of the young (MODY) (Stanescu et al., 2012).

Mutations in the *GCK* gene result in variable phenotypes ranging from mild, adult-onset HI which may be asymptomatic, to severe and diazoxide-unresponsive neonatal-onset HI requiring near-total pancreatectomy (Banerjee et al., 2019; Demirbilek and Hussain, 2017; Wabitsch et al., 2007).

On the other hand, the most common genetic variants identified in diazoxide-unresponsive cases are in the *ABCC8* and *KCNJ11* genes. Patients with biallelic inactivating mutations in *ABCC8* or *KCNJ11* often exhibit a lack of response due to severely impaired or absent channel activity (Chen et al., 2021; Brar et al., 2020; Snider et al., 2013). Homozygous recessive and compound heterozygous forms of *ABCC8* and *KCNJ11* variants typically cause severe, diazoxide-unresponsive CHI that may require intensive medical therapy or near-total pancreatectomy. Paternally-inherited recessive *ABCC8* mutations are classically associated with diazoxide-unresponsive focal lesions, although diffuse disease has been found in some cases as well (Kapoor et al., 2013). Autosomal dominant forms of *ABCC8* and *KCNJ11* mutations usually cause milder and diazoxide-responsive CHI (Shaikh et al., 2023).

Other rarer genetic variants include those involving *UCP2*, *HK1*, *PGM1*, *PMM2*, *FOXA2* and *CACNA1D* genes where there is currently insufficient data regarding diazoxide-responsiveness in these genetic variants.

Large cohort studies have consistently demonstrated that pathogenic variants in the KATP channel genes (*ABCC8* and *KCNJ11*) account for the majority of genetically confirmed CHI, particularly among diazoxide-unresponsive patients (Kapoor et al., 2013; Bellanné-Chantelot et al., 2010; Park et al., 2011). Among individuals who fail to respond to diazoxide, K_ATP_ channel mutations are identified in approximately 82%–94% of cases (Snider et al., 2013; Demirbilek et al., 2014), underscoring the central role of molecular diagnosis in therapeutic stratification. From a precision medicine perspective, understanding the genotype not only establishes etiology but meaningfully refines the probability of treatment responsiveness. Biallelic recessive K_ATP_ mutations are strongly associated with diffuse, severe disease that is frequently diazoxide-unresponsive (Snider et al., 2013; Bellanné-Chantelot et al., 2010), whereas autosomal dominant variants more often confer milder phenotypes with preserved pharmacologic responsiveness, albeit with recognized exceptions (Macmullen et al., 2011; Arya et al., 2014). Thus, inheritance pattern functions as a genomic biomarker that enriches prediction of both therapeutic response and histological subtype.

Of particular clinical relevance, paternally inherited monoallelic recessive K_ATP_ mutations are clear molecular indications of possible underlying focal CHI. In a large international cohort, monoallelic recessive variants predicted focal lesions with 97% sensitivity and 90% specificity (Snider et al., 2013), effectively transforming surgical decision-making from empiric to genomically informed. Japanese cohort data similarly demonstrated that paternally inherited monoallelic mutations were highly predictive of focal disease (Yorifuji et al., 2011). Although focal histology is confirmed in approximately 45% of patients with paternally inherited heterozygous mutations (Arya et al., 2014), molecular diagnosis has, in some series, outperformed 18F-DOPA PET imaging in predicting histological subtype (Yorifuji et al., 2011). These genotype-phenotype correlations therefore operationalize precision medicine in CHI: identification of a biallelic recessive K_ATP_ mutation substantially increases the post-test probability of diazoxide-unresponsiveness and supports early transition to alternative intense medical or surgical strategies, whereas detection of a paternal monoallelic mutation justifies expedited lesion localization through imaging and surgery. In this context, inheritance pattern is not merely descriptive but directly informs subsequent therapeutic steps and timing of intervention.

### Diazoxide-responsiveness in relation to underlying syndromes

Patients with syndromic forms of CHI often demonstrate variable response to diazoxide treatment (Zenker et al., 2023). Beckwidth-Widemann syndrome is most implicated, where patients typically present with persistent HI and these patients are often diazoxide-unresponsive with more than half requiring near-total pancreatectomy (Brar et al., 2020). Usher syndrome, a rare autosomal recessive condition involving mutations in the *ABCC8* gene, has been consistently associated with CHI, with most of these patients being diazoxide-unresponsive. Clinical features of Usher syndrome include sensorineural hearing loss and retinitis pigmentosa.

Sotos syndrome, an overgrowth disorder associated with CHI, typically demonstrates a favorable response to diazoxide. Similarly, CHI associated with Kabuki syndrome, Costello syndrome and Rubinstein-Taybi syndrome are generally diazoxide-responsive, with only a few cases requiring near-total pancreatectomy. Metabolic conditions such as congenital disorders of glycosylation and tyrosinemia have also been rarely associated with CHI which were mostly diazoxide-responsive.

### Other clinical predictors of diazoxide-responsiveness

A cohort study involving 44 patients with CHI found that patients who are diazoxide-unresponsive (n = 8) were more likely to present with hypoglycaemia within the neonatal period (62.5%), whereas the majority of diazoxide-responsive cases (n = 36) had symptoms onset between the first and sixth months of life (55.6%) (Hu et al., 2012). This suggests that a later age of onset may be positively correlated to diazoxide-responsiveness.

Birth weight (BW) is another potential clinical predictor of diazoxide-responsiveness. Infants who are LGA, defined by BW above 90th centile for gestational age, have been found to have an 8-fold increase in risk of persistent HI, and 21-fold increased risk of diazoxide-unresponsiveness. This association is particularly evident in LGA infants born to non-diabetic mothers (Falzone et al., 2020). In such cases, early genetic testing and imaging are recommended to guide timely surgical intervention, thereby minimizing the risks of recurrent hypoglycaemia and reducing hospitalization stay (States et al., 2021).

## Conclusion

This review advocates for an individualized approach to diazoxide dosing in CHI. Patient-specific factors, such as genetic variants, BW, renal function, and comorbidities, should be considered when deciding on the starting dose and titration strategies. Emerging evidence supports the use of lower starting doses of diazoxide (2–5 mg/kg/day), which may be equally effective and reduces risks of adverse effects, particularly in neonates with transient HI or known genetic variants that predicts diazoxide-responsiveness. In clinical practice, tailoring diazoxide therapy to genetic and clinical profiles could potentially enhance safety and efficacy.

Future research should focus on validating the proposed individualized, genotype-guided dosing algorithms and identifying additional clinical and molecular predictors of diazoxide-responsiveness.

## Supplementary material

SUPPLEMENTARY FIGURE S1 Literature search to understand the current knowledge of diazoxide use.

## Glossary

*ABCC8*: ATP binding cassette subfamily C member 8
AD: Autosomal dominant
ADP: Adenosine diphosphate
AgRP: Agouti-related protein
AR: Autosomal recessive
ATP: Adenosine triphosphate
BW: Birth weight
CHI: Congenital hyperinsulinism
CLD: Chronic lung disease
GDH: Glutamate dehydrogenase
GFR: Glomerular filtration rate
GIR: Glucose infusion rate
GTP: Guanosine triphosphate
HH: Hyperinsulinaemic hypoglycaemia
HI: Hyperinsulinism
IDM: Infants of diabetic mothers
IUGR: Intrauterine growth restriction
**K**_**ATP**_: ATP-sensitive potassium
*KCNJ11*: Potassium voltage-gated channel subfamily J member 11
LGA: Large for gestational age
MCT1: Monocarboxylate transport
MODY: Maturity onset diabetes of the young
NEC: Necrotizing enterocolitis
NPY: Neuropeptide Y
PDA: Patent ductus arteriosus
PH: Pulmonary hypertension
pKa: Acid dissociation constant
PSHI: Perinatal stress-induced hyperinsulinism
SGA: Small for gestational age
SUR: Sulfonylurea receptor
Tmax: Time to maximum concentration
Vd: Volume of distribution.
