# Supplementary figures and images for: From standard to individualized diazoxide therapy in congenital hyperinsulinism: a narrative review
Source: Front Pharmacol. 2026 Mar 30;17:1781424. doi: 10.3389/fphar.2026.1781424 (PMC13071010; doi:10.3389/fphar.2026.1781424)

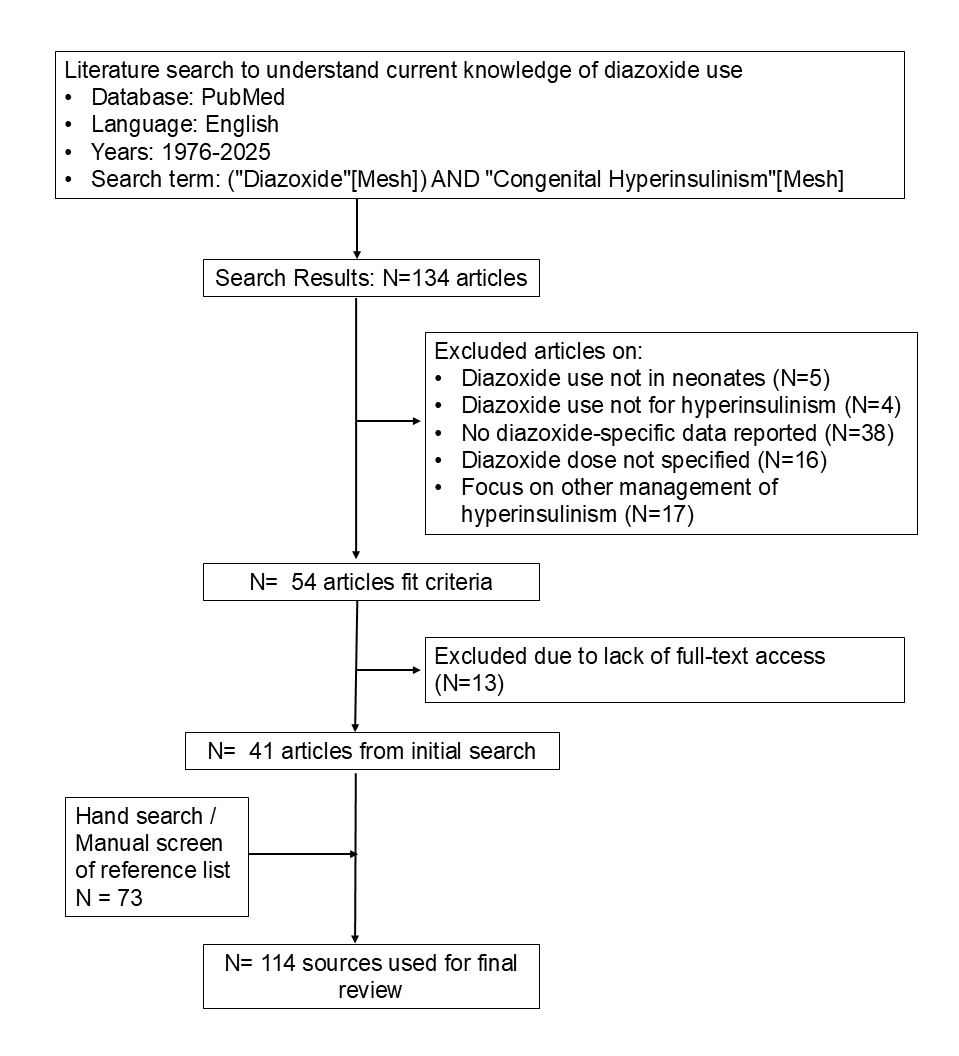

Supplement: Supplementary file 1 [file Image1.tif]
